# Simultaneously Cationic and Anionic Dyes Elimination via Magnetic Hydrochar Prepared from Copper Slag and Pinewood Sawdust

**DOI:** 10.3390/toxics11060484

**Published:** 2023-05-25

**Authors:** Huabin Wang, Yi Wu, Yi Wen, Dingxiang Chen, Jiang Pu, Yu Ding, Sailian Kong, Shuaibing Wang, Rui Xu

**Affiliations:** 1 School of Energy and Environment Science, Yunnan Normal University, Kunming 650500, China; wuyimax@foxmail.com (Y.W.); wyaquarius@foxmail.com (Y.W.); ynnuchendx@foxmail.com (D.C.); 2 Shiping Center for Rural Energy and Environment, Honghe 661400, China; 13867455685@163.com; 3 Baoshan City Longyang Rural Energy Workstation, Baoshan 678000, China; 4 Development Center for Rural Affairs of Jiangchuan District, Yuxi 651100, China; yunnanxt@foxmail.com; 5 College of Chemistry Biology and Environment, Yuxi Normal University, Yuxi 653100, China; wshuaibing@yxnu.edu.cn

**Keywords:** modified hydrochar, copper slag, dyes, simultaneous removal, adsorption mechanisms

## Abstract

In practical wastewater, cationic and anionic dyes usually coexist, while synergistic removal of these pollutants is difficult due to their relatively opposite properties. In this work, copper slag (CS) modified hydrochar (CSHC) was designed as functional material by the one-pot method. Based on characterizations, the Fe species in CS can be converted to zero-valent iron and loaded onto a hydrochar substrate. The CSHC exhibited efficient removal rates for both cationic dyes (methylene blue, MB) and anionic dyes (methyl orange, MO), with a maximum capacity of 278.21 and 357.02 mg·g^−1^, respectively, which was significantly higher than that of unmodified ones. The surface interactions of MB and MO between CSHC were mimicked by the Langmuir model and the pseudo-second-order model. In addition, the magnetic properties of CSHC were also observed, and the good magnetic properties enabled the adsorbent to be quickly separated from the solution with the help of magnets. The adsorption mechanisms include pore filling, complexation, precipitation, and electrostatic attraction. Moreover, the recycling experiments demonstrated the potential regenerative performance of CSHC. All these results shed light on the co-removal of cationic and anionic contaminates via these industrial by-products derived from environmental remediation materials.

## 1. Introduction

With the rapid development of social industry and textile scale, the immature technology of dye wastewater treatment systems leads to a large number of compounds and intermediates participating in the process of dye production and dyeing. As the wastewater is discharged into external water bodies, the treatment capacity of dye wastewater also decreases [1,2]. The ecological and environmental problems caused by printing and dyeing wastewater are inevitable and ubiquitous in daily life [3]. The printing and dyeing wastewater is a typical high-pollution wastewater, which is characterized by large discharge, high chroma, high salt concentration, strong acid, strong alkali, strong resistance to microorganisms, and so on [4,5]. Some organic compounds and toxic heavy metals pose carcinogenic, teratogenic, and mutagenic risks to humans [6]. In addition, due to the variety of dyes and complex industrial production, there are always different kinds of dye residues in the wastewater, that is, anionic dyes and cationic dyes always coexist [7]. However, the physical and chemical properties of anionic and cationic dyes are basically opposite, and it is usually difficult to remove both dyes at the same time.

Coagulation and flocculation, precipitation, microbial, electrolysis, adsorption, and membrane separation are currently commonly used to remove dyes [8,9]. However, each method has its advantages and disadvantages. For example, although the microbial method can utilize cheap renewable resources, the microbial growth and culture path is long, so it is easy to produce more by-products in the follow-up experiment, and the reaction cycle is long. The membrane separation method is simple to operate and has little pollution, but with the increase of the use time, the surface of the membrane will be polluted, which reduces the performance. Moreover, this method only separates and transfers the pollutants and cannot fundamentally achieve adsorption. According to previous studies, the adsorption method still has advantages, which are far ahead of other methods in terms of application value and actual removal effect. At the same time, it has the advantages of good removal effect, short time, low cost, simple operation, good cycling performance, and not easy to cause harm to the environment. The high-efficiency adsorbent for printing and dyeing wastewater is the core issue of the adsorption.

In recent years, zero-valent iron (ZVI) nanoparticles with abundant REDOX active sites have received a lot of focus [10]. However, ZVI tends to accumulate in solution, which reduces its reactivity. Therefore, some porous materials are used to support ZVI to improve the stability of ZVI, such as activated carbon, zeolite, biochar, and so on. Nevertheless, the synthesis of ZVI composites is high-cost and low-load, and it is necessary to optimize ZVI composites. HC is a product prepared under high pressure and low temperature, with rich functional groups and porous structure [11,12], which shows great potential in removing pollution [13,14]. Hence, HC seems to be an ideal material to load ZVI. At present, the main sources of ZVI are K_3_[Fe(C_2_O_4_)]·3H_2_O [15] and FeCl_3_·6H_2_O [16,17], reduced iron powder, and iron ores, which mainly includes limonite, siderite, and magnetite [18]. These materials have the disadvantages of complex preparation methods and high cost [19], although they have the advantages of environmental protection, economy, and efficient use [20,21]. The composite material of hydrochar and ZVI prepared with the above modifiers is at variance with the concept of sustainable development. Therefore, it is urgent to find new modified materials.

The accumulated stock of copper slag (CS) in China has reached 120 million tons [22], but the actual utilization of CS is rare. The iron in CS mainly exists in the form of weakly magnetic iron, and the effect of metal recovery by traditional methods is ineffective. The long-term and large accumulation of CS not only causes land occupation but also damages the soil and water environment [23]. Iron, copper, and other valuable metals are prevalent in CS, and it is worth noting that the iron content in CS (Fe% = 35%) is significantly greater than that recovered from iron ore (Fe% > 27%) [24]. Our research group has used CS as the modified material to prepare the relevant functional bio-adsorbent via the hydrothermal reduction method to remove heavy metal (Selenium) from the solution, and the absorption efficiency of the bio-adsorbent was great. However, whether the magnetic hydrochar modified by CS can be used in dye wastewater treatment and whether it can achieve cationic and anionic dyes elimination simultaneously remain to be studied. Moreover, the adsorption mechanism and industrial feasibility also need to be explored.

Therefore, in this research, we prepared magnetic hydrochar modified by CS and explored the removal efficiency of cationic dyes (methylene blue, MB) and anionic dyes (methyl orange, MO) in printing wastewater. The intention of this work was to obtain adsorbent magnetic hydrochar modified by CS via the hydrothermal reduction called CSHC and use CSHC for the adsorption of dyes. In this study, we believe that pine sawdust is used as the precursor to prepare hydrochar, and CS was innovatively modified with the help of high cellulose content and rich lignin in the structure. The combination of the two can not only effectively expand the specific surface area but the pore system of adsorbent materials. Moreover, the ZVI reduced in the preparation process could also be effectively attached to the hydrochar, thus providing a greater possibility for the adsorption of target pollutants. Therefore, the modified hydrochar was prepared by the one-pot method using CS as an iron source and pine sawdust as a carrier to explore its removal efficiency of cationic dyes (methylene blue, MB) and anionic dyes (methyl orange, MO) in printing and dyeing wastewater is a promising solution. Modified hydrochar (CSHC) was prepared from copper slag and pine sawdust as a high-performance adsorbent and applied to the removal of MB and MO from printing and dyeing wastewater. Various characterization methods were used to investigate the physical and chemical properties of the prepared CSHCS before and after modification. The simultaneous removal of MB and MO was tested by batch adsorption experiments. Thermodynamic analysis and kinetic model were used to illustrate the surface interaction and adsorption types, respectively. The reusability of the composite functional environmental protection material was evaluated by a recycling test. All this work could provide a promising and potential solution for CS reutilization and wastewater purification.

## 2. Resources and Techniques

### 2.1. Research Materials

The CS was obtained from Xuanwei Phoenix Steel Co., Ltd., from Qujing City, China. The pinewood sawdust was collected from Kunming City, China. The methylene blue (C_16_H_18_ClN_3_S, solubility in water: 40 g·L^−1^ at 25 °C, molar mass: 373.9 g·mol^−1^), methyl orange (C_14_H_14_N_3_NaO_3_S, solubility in water: 200 mg·L^−1^ at 25 °C, molar mass: 327.3 g·mol^−1^), NaOH, and other reagents were provided from Beijing Chemical Reagent Factory. The pinewood SD was rinsed with distilled water and dried at 80 °C, and then the dried pinewood sawdust was crushed and passed through 80 mesh.

### 2.2. Adsorbent Preparation Methods

**Scheme 1:**
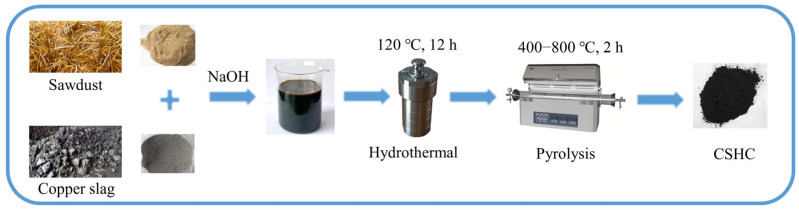
Flowchart of preparation of CSHC.

The preparation of CSHC is shown in Scheme 1 [25]. Firstly, weighed CS (4 g) and CS (4 g) were mixed. Secondly, the mixture was steeped in NaOH solution (200 mg·L^−1^, 40 mL) and distilled water (60 mL) in order to reduce the crystallinity of cellulose in SD and improve the mixing degree, and then it was mixed by ultrasonic oscillation (25 °C, 0.5 h) and stirred by a magnetic stirring apparatus (25 °C, 2 h). Thirdly, the mixture had a hydrothermal reaction (120 °C, 10 h). Finally, the dried slurry was placed in a tube furnace and underwent a pyrolysis reaction (heating rate was 10 °C·min^−1^, pyrolysis temperature was 400, 600, or 800 °C, and holding time was 2 h, N_2_ protection). The three samples were washed with deionized water three times and were labeled CSHC400, CSHC600, and CSHC800, respectively. In addition, 8.0 g SD and 8.0 g CS were weighed and pyrolyzed (heating rate was 10 °C·min^−1^, pyrolysis temperature was 600 °C, and holding time was 2 h, N_2_ protection), respectively, and the products were labeled HC600 and CS600.

### 2.3. Analysis Methods

The surface functional groups of the hydrochar were measured with Fourier transform infrared spectroscopy (FTIR, Spectrum Two, Perkin-Elmer, Waltham, MA, USA) using the KBr disk technique. Scanning electron microscopy (SEM, FEI Quanta 200FEG, Thermo Scientific Quanta, Hillsboro, OR, USA) was used to detect the surface morphology. The specific surface area (SSA) of biochar was detected by N_2_ adsorption isotherms at 77 K using a Micropore Analyzer (ASAP 2460, Micrometrics, Norcross, GE, USA). The crystal structure was characterized by X-ray diffractometry (XRD, D8 Advance Sox-l, Bruker Co., Billerica, MA, USA) with Cu K-alpha radiation at 40 kV (λ = 0.15418 nm). An X-ray photoelectron spectrometer (XPS, ESCALAB 250Xi, Thermo Fisher Scientific, Waltham, MA, USA) was used to characterize the surface composition and chemical state of the modified bone hydrochar before and after the adsorption of the organic dyes.

### 2.4. Batch Experiment

All sorption experiments were performed in a 10 mL centrifuge tube with varied environmental factors at optimum operational parameters. The initial solution pH was adjusted with trace amounts of 0.001–0.1 mol·L^−1^ HCl/NaOH solutions. The adsorption kinetics were investigated by dispersing 10.0 mg of adsorbent into 10 mL of aqueous MB and MO solutions containing different initial concentrations (i.e., 50 or 500 mg·L^−1^) at pH = 12 for MB and pH = 3 for MO, T = 318 K. The adsorption isotherm experiments were carried out by vigorously shaking 10 mL of a solution containing varied levels of organic dyes (MB and MO from 25 to 500 mg·L^−1^) mixed with 10.0 mg of CSHC for 3 h to reach equilibrium at pH = 12.0 and pH = 3.0, respectively, T = 318 K. 

Adsorption experiments were carried out in an orbital shaker (HNY-100B, Honour instrument shaker, Tianjin, China) at 120 rpm·min^−1^ for 3 h to ensure that the sorption reached equilibrium. The supernatants were then filtered with a 0.22 μm filter membrane, followed by determining the MB and MO concentrations (*C_e_*, mg·L^−1^) with an Ultraviolet-visible spectrophotometer (UV-722, Evolution 201 & 220, Thermo Fisher Scientific, Waltham, MA, USA) to measure the quantitative absorbance of these organic dyes under the influence of an adsorbent. Then, the MB and MO adsorption capacities of the adsorbent materials were computed. The MB was centered at a wavelength of 663 nm [26], and the MO was centered at a wavelength of 463 nm [27]. According to Formulas (1) and (2), the adsorption capacity *q_e_* (mg·g^−1^) and removal rate can be calculated, respectively. The calculation formula of the Langmuir model and Freundlich model was shown as Formulas (3) and (4). Additionally, Formulas (5) and (6) provided the expressions for the pseudo-first-order model and pseudo-second-order model. In order to ensure the uniformity of measurement and accuracy of dates, all experiments were repeated three times.

(1) $$q_{e}=\frac{(C_{0}-C_{e})\times V}{W}$$

(2) $$Removal\;rate=\frac{C_{0}-C_{e}}{C_{0}}\times 100\%$$

where *C_0_* (mg·L^−1^) was the initial concentration of dye, *C_e_* (mg·L^−1^) was the concentration of dye solutions after adsorption, *V* (L) was the volume, and *W* (g) was the quantity of the adsorbent.

(3) $$\;\mathrm{Langmuir}\;\mathrm{model}:\;q_{e}=\frac{q_{m}\;\cdot K_{L}\;\cdot C_{e}}{1+K_{L}\;\cdot C_{e}}\;\;\;$$

(4) $$\mathrm{Freundlich}\;\mathrm{model}:\;q_{e}=K_{L}\cdot C_{e}^{\frac{1}{nF}}\;\;\;$$

where *C_e_* (mg·L^−1^) was the action’s equilibrium content, *q_e_* (mg·g^−1^) was the target pollutant’s absorption capacity, *K_L_* (L·mg^−1^) was the constant in the Langmuir model, *K_F_* (mg^1−n^ L^n^·g^−1^) was the constant in the Freundlich model, and *q_m_* (mg·g^−1^) was the highest adsorbent capacity.

(5) $$\mathrm{Pseudo-first-order}\;\mathrm{model}:\;q_{t}=q_{e}\left(1-e^{-k_{1}t}\right)$$

(6) $$\mathrm{Pseudo-second-order}\;\mathrm{model}:\;q_{t}=\frac{q_{e}^{2}k_{2}t}{1+q_{e}k_{2t}}\;\;\;\;\;\;\;\;$$

The quantities of MB and MO that were adsorbed onto the CSHC600 at equilibrium and time t, respectively, are denoted by the variables *q_e_* and *q_t_* in these formulations, whereas *k*_1_ (min^−1^) and *k*_2_ (g·(mg min)^−1^) were the constants for the pseudo-first-order and pseudo-second-order models, respectively. The fitting results are shown in Table 1. The final fitting results showed that during the adsorption of MB, the R^2^ of the pseudo-second-order model was superior to that of the pseudo-first-order model, demonstrating that chemical adsorption dominated. Moreover, *k*_2_ on MB (2.58 × 10^−2^) was larger than *k*_2_ on MO (1.87 × 10^−4^), indicating that the equilibrium time of adsorption of MB by CSHC600 was shorter than that of MO.

## 3. Findings and Discussion

### 3.1. Adsorbent Material Characterization

**Figure 1:**
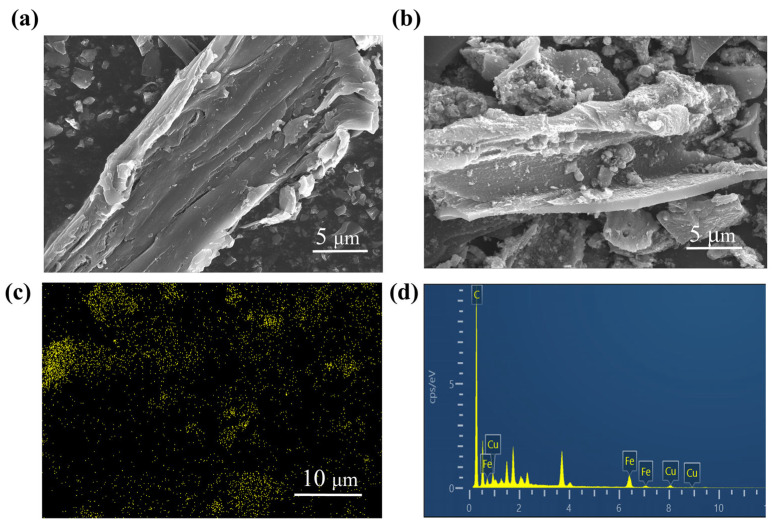
SEM images of HC600 (**a**) and SEM-EDS images of CSHC600 (**b**–**d**).

SEM directly displayed the morphologic characteristics of HC composites. The morphologies of unmodified HC600 and CSHC600 are shown in Figure 1. It was obvious to observe the different morphologies of HC600 and CSHC600. Compared with CSHC600, HC600 had a more smooth surface and dense structure with the unobvious distribution of micropores [28]. The edges of HC600 were mainly broken sheets, and a few fibrous structures could be detected, which was likely due to the pyrolysis of structural fibers in the SD [29]. There were metallic luster and white particles on the surface of CSHC600, which was most likely ZVI. The existence of ZVI could be attributed to the magnetic nanoparticles (Fe_3_O_4_) [30,31] and demonstrated that zero-valent iron effectively bonded to the surface of hydrochar [32]. In addition, some pores with different sizes were formed on the surface of CSHC600, which showed random and uneven characteristics in disorganized directions, a large number of irregular agglomeration spherical particles were attached, and a small number of micro and macro cracks appeared around the agglomeration particles, which may be the cross-linking process of multi-pore development [26]. It helped the dispersion and adsorption of dye. Compared with HC600, it was also noted that the pore diameters of CSHC600 were significantly reduced (Figure 1b). This phenomenon was due to the introduction of magnetite modifications, which blocked the pore.

**Figure 2:**
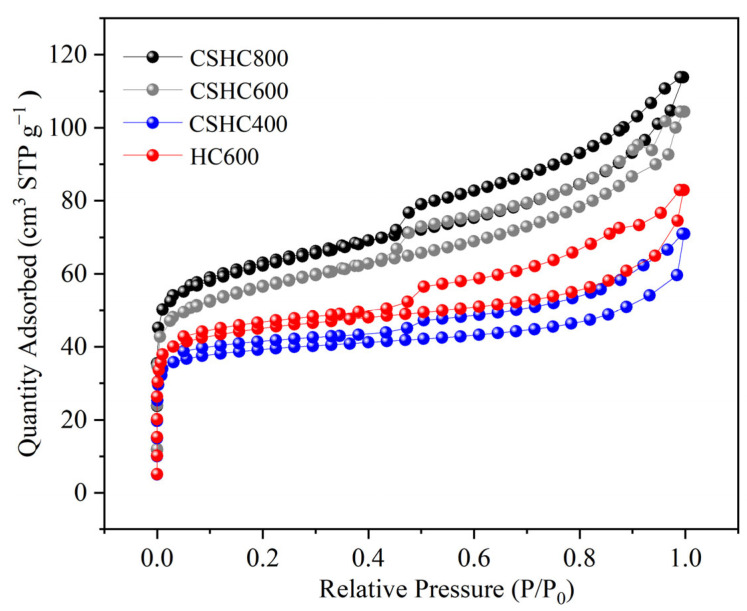
N_2_ adsorption/desorption isotherms of four activated carbons.

According to Figure 2, based on the IUPAC classification, CSHC is a typical Type I adsorption isotherm. When P/P^0^ ≤ 0.48, adsorption isotherms coincide with desorption isotherms, indicating that activated carbon has a microporous structure. With the increase of pressure, capillary condensation occurs in the pore structure, and the desorption rate of N_2_ is higher than the adsorption rate, and H4-type hysteresis rings are produced, indicating the existence of a mesoporous structure. Such microporous mesopores not only provide more adsorption sites but also improve the adsorption rate. The formation of pores is caused by the dehydration of hydrochar. Surface materials begin to aggregate and form granular microcarbon balls. There are pores of different sizes inside and between the particles. Table 1 shows that the specific surface area of CSHC600 and HC600 are 186.40 m^2^·g^−1^ and 122.33 m^2^·g^−1^, respectively, and the total pore volume is 0.15 m^3^·g^−1^ and 0.12 m^3^·g^−1^, indicating that the pore structure and S_BET_ are improved by about 1.52 times after CS modification. V_tot_ improved by about 1.25 times. This shows that CS-modified hydrochar can be a very good preparation of high specific surface area adsorbent.

**Figure 3:**
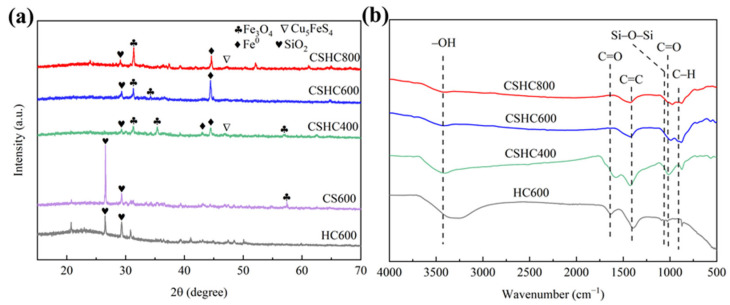
(**a,b**) XRD diffractograms and FTIR spectra of CSHC, CS600, and HC600.

The XRD diffractograms of HC600 and CS600 are shown in Figure 3. There was an intense peak corresponding to SiO_2_ at 2θ of 26.66°, and there were diffraction peaks of CaCO_3_ at 2θ of 20.95° and 29.56°. In the XRD diffractograms of CSHC, there were diffraction peaks of Fe^0^ and Fe_3_O_4_ at 2θ of 44.57°, 35.47°, and 57.54°, respectively. The diffraction peak at 2θ = 46.81° represented Cu_5_FeS_4_, and the appearance of Cu_5_FeS_4_ may be the result of a high-temperature vulcanization reaction between copper, iron, and residual sulfur in CS during the pyrolysis process [33]. Compared HC600 to CSHC600, Fe^0^ and Fe_3_O_4_ were successfully attached to the surface of the modified hydrochar [34]. In addition, when the temperature rose, the peak intensity of Fe^0^ rose. This was because more reducing gases were created at high temperatures, which reduced Fe^2+^ and Fe^3+^ into Fe^0^.

In the FTIR spectra (Figure 2b), the wide peaks at 3435 cm^−1^ and 877 cm^−1^ [35] represented the O–H and C–H, and the oscillation peak at 1647 cm^−1^ and 1008 cm^−1^ represented C=O [36,37], and the peak at 1065 cm^−1^ represented Si–O–Si [38]. The FTIR spectra showed the presence of functional groups such as –OH (3429 cm^−1^), C=O (1008 cm^−1^), C–H (1647 cm^−1^), C=C (1426 cm^−1^), and so on in these adsorbents.

In addition, it was observed that the oxygen-rich functional groups in the modified hydrochar prepared by pyrolysis at 600 °C and 800 °C were more than adsorbent prepared by pyrolysis at 400 °C. This indicated that pyrolysis at 600 °C stimulated and retained oxygen-rich functional groups and aromatic substances better [38], and thus CSHC600 had a better effect on the adsorption of MB and MO.

**Figure 4:**
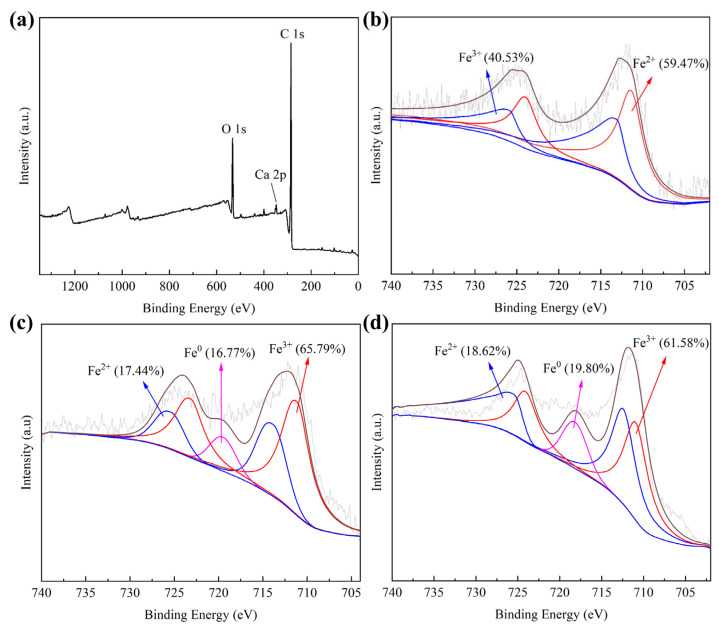
The full spectrum of CSHC600 (**a**) and XPS images of CSHC400 (**b**), CSHC600 (**c**), and CSHC800 (**d**).

The XPS analysis is shown in Figure 4. The binding energy at 288.1 eV and 529.1 eV represented C 1s and O 1s, respectively, and the binding energy at 348.3 eV represented Ca 2p. The peaks at 710.13 eV and 725.14 eV represented Fe^2+^, moreover, the peaks at 711.26 eV and 727.24 eV represented Fe^3+^. The peaks at 720.10 eV represented Fe^0^, indicating that Fe^0^ was successfully loaded onto HC [39,40].

**Figure 5:**
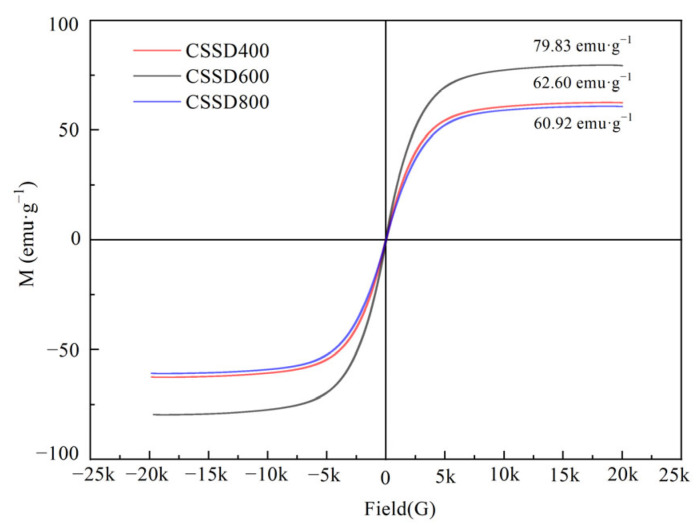
VSM spectra of CS600, CSHC400, CSHC600, and CSHC800.

The magnetic properties of CSHC400, CSHC600, and CSHC800 were determined by a vibrating sample magnetometer with a pole diameter of 5 cm under the condition of 300 K. The magnetic effect of zero-valent iron modified hydrochar was prepared from copper residue. The saturation magnetic strength of CSHC600 was 79.83 emu·g^−1^, while that of CSHC400 and CSHC800 were 62.60 emu·g^−1^ and 60.92 emu·g^−1^, respectively (Figure 5). Thus, the high-temperature pyrolysis magnetized the hydrochar [41], but the magnetization did not change with temperature. The intensity of magnetization was related to the generation of Fe_3_O_4_ during pyrolysis, and Fe_3_O_4_ had a high conductivity [42]. The adsorption properties were usually due to the electron transfer of ferromagnetism (ferrite magnets) between Fe^2+^ and Fe^3+^. A secondary phase transition occurred above the Curie temperature to a paramagnetic substance. Due to the Curie temperature of Fe_3_O_4_ being 585 °C, the pyrolytic temperature of 600 °C can oxidize the iron part of CS to Fe_3_O_4_ to the greatest extent. The stronger the magnetic properties of the adsorbent, the more favorable the magnetization separation between the adsorbent and the dyeing wastewater [43]. Under the attraction of a permanent magnet, the adsorbent can be quickly separated from the adsorption liquid. Considering the adsorption capacity and magnetic properties, CSHC600 was considered the optimal one for further analysis.

### 3.2. Analysis of Batch Experiments

#### 3.2.1. Effect of pH

**Figure 6:**
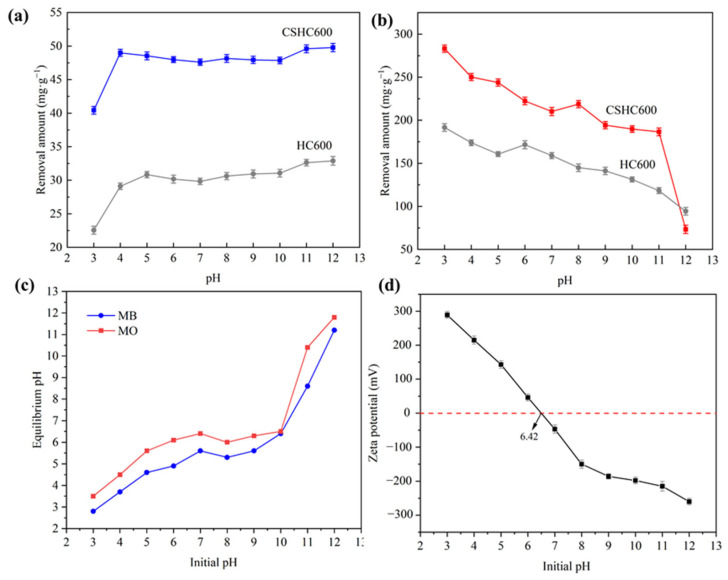
MB (**a**) and MO (**b**) were adsorbed onto CSHC600 and HC600 at various pH levels; Solution pH after adsorption of MB and MO (**c**); Zeta potential determination (**d**). Experiment conditions: [dosage = 1 g·L^−1^], [temperature = 318 K], [MB = 50 mg·L^−1^], [MO = 500 mg·L^−1^], [reaction time = 3 h].

Figure 6 shows the effect of pH value on the adsorption capacity of CSHC600. The initial concentration of MB was 50 mg·L^−1^, the initial concentration of MO was 500 mg·L^−1^, and the pH of the solution was 3–12. When pH > 3, the adsorption capacity of CSHS600 for MB was high, but the overall trend was stable. When pH = 10, the adsorption capacity increased slightly. The results showed that CSHS600 can remove MB over a wide pH range. For MO, the maximum adsorption capacity appealed when pH = 3, but the adsorption capacity evidently decreased with the increase of pH value. This was probably due to pHpzc 6.42. The positive charge of protonation of functional groups on the surface of CSHC600 led to positive zeta potential, and the anionic dye MO can be adsorbed by electrostatic adsorption. As can be seen from Figure 6, the adsorbent had a strong adsorption capacity for MB under an alkaline environment and MO under an acidic environment [44]. According to previous studies, the adsorption effect of CS-derived zero-valent iron-modified hydrochars on MB and MO solutions varies significantly at different pH levels [45]. In addition, the pH of the adsorbed solution was measured, and it was found that for MB, the pH level varied from 2.8 to 11.2. Similarly, the pH of MO varies from 3.5 to 11.8. The reason for this result may be that electrostatic attraction neutralizes part of the charge in the adsorption process, making the solution nearly neutral. However, the adsorbent material itself shows alkalinity, so most of the solution after adsorption is weak alkalinity, while the individual shows strong alkalinity.

#### 3.2.2. Analysis of Isothermal Adsorption

**Figure 7:**
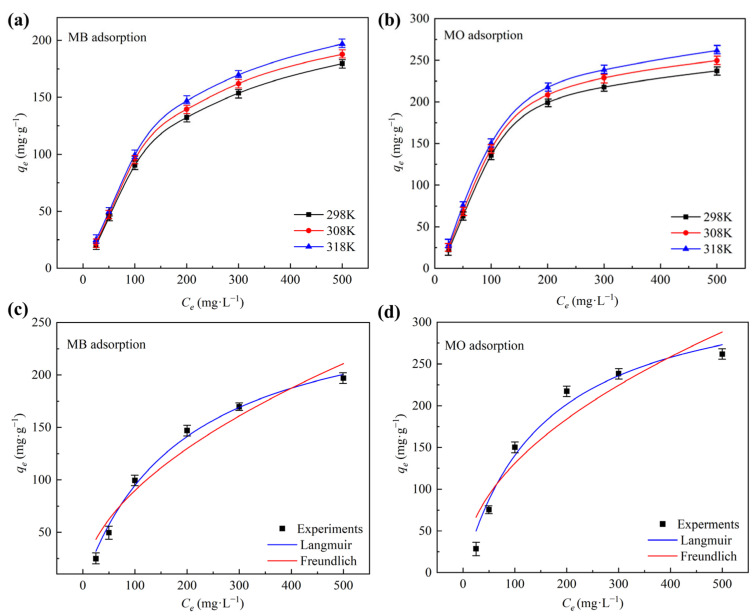
The MB (**a**) and MO (**b**) removal capacities of the isotherms, thermodynamics (**c**); Fitted curves of MB (**c**) and MO (**d**) for the Langmuir and Freundlich isotherms. Experiment conditions: [dosage = 1 g·L^−1^], [temperature = 318 K], [MB = 50 mg·g^−1^], [MO = 500 mg·g^−1^], [reaction time = 3 h].

The adsorption efficiency of CSHC600 on MB and MO at various initial concentrations are shown in Figure 7c,d, and the similarities between MB and MO were obvious. The adsorption capacity gradually rose as the pollutant concentration rose. The adsorption capacity of CSHC600 tended to be steady when the concentration rose to a certain point. The experimental dates were fitted by the Langmuir and Freundlich models [46], and the results are shown in Table 2. For the adsorption MB by CSHC600, the R^2^ of the Langmuir model was considerably higher (0.99) than that of the Freundlich model (0.94). For the adsorption MO by CSHC600, R^2^ values were 0.97 and 0.88 for the Langmuir and Freundlich models, respectively. Thus, the MB and MO absorption process was better fitted by the Langmuir model [47]. The statement of Langmuir has four basic assumptions. There is no interaction between substrates, there is no adsorption site with the same energy, all adsorption sites are equivalent, and the substrate is uniformly distributed on the surface of the adsorbent monolayer film [48,49]. In other words, the adsorption preponderance of MB and MO by CSHC600 was mediated by chemisorption on a single molecular layer [50,51]. CSHC600 was an effective MB and MO adsorbent, which showed the best effect [52], as its greatest adsorption capacity respectively reached 278.21 mg·g^−1^ and 357.02 mg·g^−1^ for MB and MO (Table 2), which compared with other research data (Table 3). These results show that CSHC600 was an effective substance for removing these two kinds of organic dyes.

#### 3.2.3. Adsorption Kinetics

**Figure 8:**
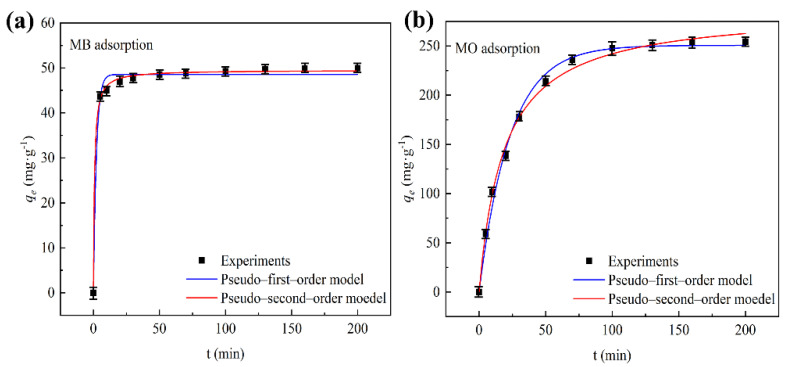
The adsorption kinetics of MB (**a**) and MO (**b**). Fitting of adsorption kinetics by linear relations of pseudo-first-order and pseudo-second-order models.

Response time was also a key parameter in adsorption, which reflected the adsorption speed. Response time contributed to the efficient removal of pollutants and provided more valuable information for the adsorption process. According to the existing experimental data, CSHC600 had the best adsorption capacities for MB and MO when pH = 12 and pH = 3, respectively. The results of the fitting are displayed in Figure 8a,b. The adsorption equilibrium for MO was 130 min, and the adsorption equilibrium for MB was 30 min. The adsorption capacity of MO and MB increased rapidly during the early stages of adsorption, increased slowly, and ultimately tended to a stable state. MB and MO rapidly diffused from the solution to the surface of the adsorbent material, after that, the adsorption tended to be slow. There may have been a lot of adsorption active sites on the surface of CSHC600, but when the reaction was going on, these active sites gradually filled with small molecules such as MO and MB, which weakened the adsorption. 

#### 3.2.4. Thermodynamic analysis

Thermodynamic analysis was carried out by evaluating the entropy and energy changes in the adsorption process. The formula for figuring out the Gibbs free energy and other factors during the adsorption process is shown in Formulas (7) and (8).

(7) $$\ln\left(\frac{q_{m}}{C_{e}}\right)=\frac{\Delta S^{0}}{R}-\frac{\Delta H^{0}}{RT}\;$$

(8) $$\Delta G^{0}=\Delta H^{0}-T\Delta S^{0}$$

where ∆*G*^0^ represented the change in free energy, ∆*S*^0^ represented the change in entropy, and ∆*H*^0^ represented the change in enthalpy. The isothermal adsorption data were used to calculate $\ln\left(\frac{q_{m}}{C_{e}}\right)$, which had been given a value of 8.314 J·(mol·K)^−1^ [58], where T was the temperature and R was the universal gas constant.

Table 2 showed that ∆*G^0^* adsorbing MB by CSHC600 at 318 K was −4.10 KJ·mol^−1^, while that of MO was −6.02 KJ·mol^−1^. Both ∆*G^0^* values were negative when adsorbing MB and MO, indicating that the adsorption reactions may be totally irreversible [59]. The ∆*H^0^* was positive, indicating the reactions were endothermic.

### 3.3. Regeneration Experiments

**Figure 9:**
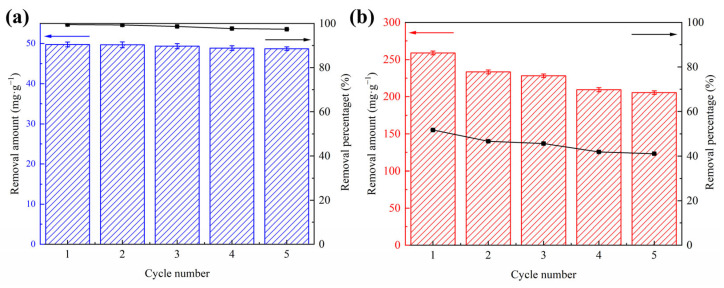
Adsorption of MB (**a**) and MO (**b**) using CSHC600 as an adsorbent that is reusable. Experiment conditions: [dosage = 1 g·L^−1^], [temperature = 318 K], [MB = 50 mg·g^−1^], [MO = 500 mg·g^−1^], [reaction time = 3 h].

**Figure 10:**
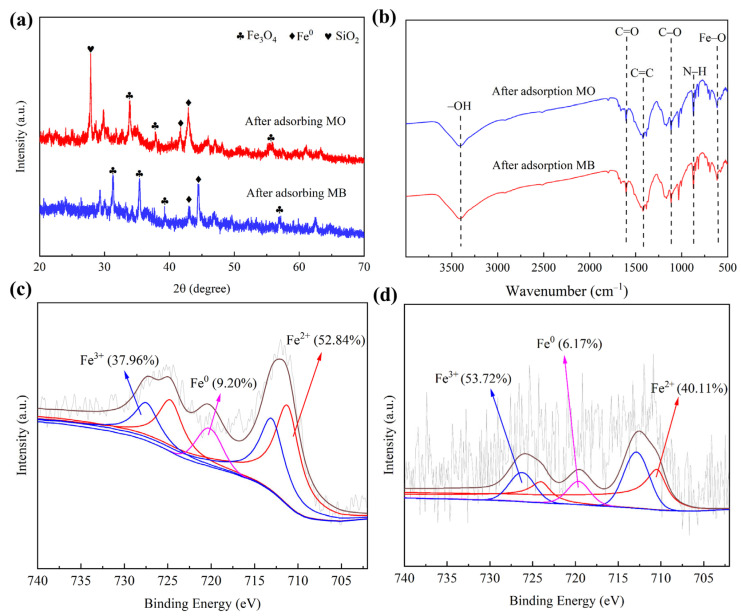
The images of XRD, FTIR, and XPS of CSHC600 after adsorption. (**a**) XRD diffractograms, (**b**) FTIR spectra, (**c**) XPS of CSHC600 adsorption of MB, (**d**) XPS of CSHC600 adsorption of MO.

CSHC600 was a reusable adsorbent (Figure 9). Solvent desorption was used to evaluate the reproducibility of CSHC. The main method was achieved through five continuous adsorption-desorption processes [47]. To achieve pH neutrality, the attachments of the adsorbent were eluted, and the residue was repeatedly rinsed with deionized water. The recovered adsorbent was dried in a vacuum drying oven at 60 °C. To explore regeneration capabilities, the adsorption was placed into the same adsorption circumstances as previously, and the adsorption-desorption cycle was carried out five times. By using CSHC600, MB (50 mg·L^−1^) and MO (500 mg·L^−1^) can be reused with ease. As shown in Figure 10, MB could almost be desorbed completely. The removal rate of CSHC600 on MB was still achieved at 97% after five cycles. At pH = 3, the removal rate of MO by CSHC600 was 51% in the first cycle and more than 41% in the fifth cycle. The removal rates of CSHC600 for MB and MO were great even after five cycles. The decrease in adsorption efficiency may be related to the fact that some original adsorption sites of CSHC600 were occupied and did not regenerate after the preliminary test. The removal rates of CSHC600 for MB and MO were great even after five cycles. The outcomes demonstrate that CSHC600 was simple to recycle and has strong adsorption capability. 

### 3.4. Mechanisms

**Figure 11:**
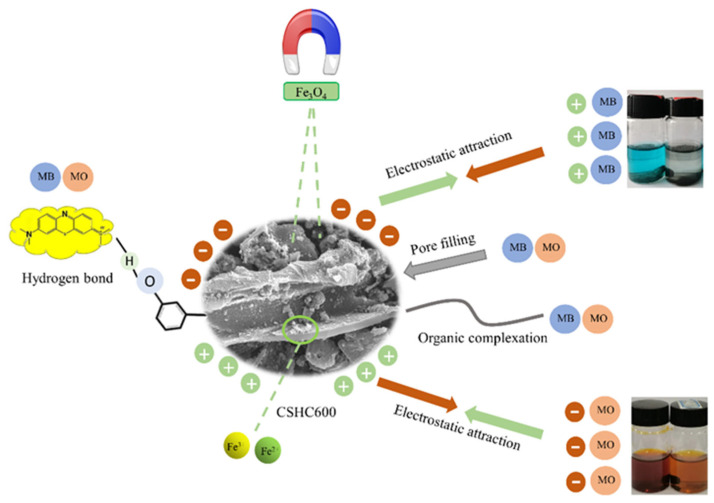
Adsorption mechanism of MO and MB by CSHC.

The removal mechanisms of MB and MO were studied. The SEM images showed that CSHC600 had a smooth surface and uniform pore distribution. In addition, the crystal structure of the adsorbent was discovered. The FTIR spectra after adsorption (Figure 10) showed that the interaction of these functional groups on the surface of CSHC600 with MB and MO, for example, –OH and –COOH functional groups shifted, the strength of C–C functional groups decreased, and additionally C–O and –COOH participated in the reaction. This indicated that hydrogen bonding existed in the adsorption process of CSHC600 [60]. By comparing the XRD diffractograms of CSHC before and after adsorption, it can be seen that Fe^0^ decreased obviously after adsorption. It was also clear that the peak of Fe^0^ significantly weakened after adsorption, which may be because ZVI loaded on CSHC600 reduced mineralized MB and MO. Eventually, the dye was degraded to small molecules such as CO_2_ and H_2_O, while ZVI was oxidized to Fe^2+^ and Fe^3+^. As MO was a typical anionic compound, the positive and negative charges between Fe^2+^, Fe^3+^, and MO attracted each other, which attached MO to the surface of the adsorbent. According to the XPS of CSHC before and after adsorption, the peak of Fe^3+^ increased significantly after adsorption. A large amount of Fe^3+^ flocculated with the free –OH in solution to form Fe(OH)_3_. Precipitation was the main mechanism of dye adsorption by hydrochar. In the same way, HC was usually alkaline and carried a negative charge, which can be well combined with cationic dye (MB) via complexation (Figure 11).

## 4. Conclusions

In this work, the green construction of CSHC was realized by the hydrothermal method with pine sawdust as a carrier and copper slag as an iron source. A variety of characterization methods have demonstrated the successful loading of ZVI. The adsorption process of CSHC600 on MB and MO was fitted by the pseudo-second-order kinetic equation and Langmuir model, respectively. The adsorption process is mainly controlled by chemisorption, and the maximum adsorption capacity is 278.21 mg·g^−1^ and 357.02 mg·g^−1^, respectively. CSHC600 has good recovery performance. The experimental results prove the feasibility of copper slag and pine chip adsorbent for purifying industrial wastewater containing cationic dyes and anionic dyes.

## Figures and Tables

**Table 1:** Pore structure analysis of four activated carbons.

Sample	Specific Area (m^2^·g^−1^)	Pore Volume (m^3^·g^−1^)	Pore Size (Å)
CSHC800	201.33	0.16	27.15
CSHC600	186.40	0.15	28.37
CSHC400	159.71	0.13	25.32
HC600	122.33	0.12	20.35

**Table 2:** Adsorption isotherms, kinetics, and related factors summarized.

Isotherms	Target	Experimental Data	Parameters		R^2^
Langmuir			*q_m_* (mg·g^−1^)	*K_L_* (L·mg^−1^)	
	MB	196.89	278.21	0.005	0.99
	MO	261.78	357.02	0.006	0.97
Freundlich			*K_F_* (mg^1−n^L^n^·g^−1^)	1/*n*	
	MB	196.89	7.80	0.53	0.94
	MO	261.78	13.68	0.49	0.88
Kinetics	Target	Experimental data	Parameters		
Pseudo-first-order			*q_e_* (mg·g^−1^)	*k_1_* (min^−1^)	
	MB	49.95	48.56	0.43	0.99
	MO	254.38	250.65	0.04	0.99
Pseudo-second-order			*q_e_* (mg·g^−1^)	*k_2_* (g·(mg·min)^−1^)	
	MB	49.95	49.52	2.58 × 10^−2^	0.99
	MO	254.38	287.14	1.87 × 10^−4^	0.99
Thermodynamics	*T* (K)	∆*G^0^* (KJ·mol^−1^)	∆*S^0^* (KJ·(mol·K)^−1^)	∆*H^0^* (KJ·(mol·K)^−1^)	
MB	298	−2.74			
	308	−3.68			
	318	−4.10	1.96 × 10^−2^	2.13	
MO	298	−4.85			
	308	−5.78			
	318	−6.02	3.11 × 10^−2^	3.87	

**Table 3:** Comparison of different magnetic hydrochar or biomass-based adsorbents’ abilities to bind MB and MO.

Sample	Dosage (g·L^−1^)	pH	Initial Concentration (mg·L^−1^)	Capacity (mg·g^−1^)		
				MB	MO	
Fe_3_O_4_-PAMH	0.8	11 for MB	100	148.84	202.02	[53]
		5 for MO				
BM	0.5	7	50	2	41.49	[54]
NFGPBC	0.7	7	50	67.54	-	[55]
Asph-Al	10	3	20	-	41.25	[56]
FE-LB	2	4	200	180.00	-	[57]
CSHC600	1	12 for MB	50 for MB	49.58	261.78	This study
		3 for MO	500 for MO			

## Data Availability

Not applicable.
